# Quantitative Assessment of the Influence of *Rhizoma Zingiberis* on the Level of Aconitine in Rat Gut Sacs and Qualitative Analysis of the Major Influencing Components of *Rhizoma Zingiberis* on Aconitine Using UPLC/MS

**DOI:** 10.1371/journal.pone.0124110

**Published:** 2015-05-15

**Authors:** Yang Xin, Shuying Liu

**Affiliations:** 1 College of Chemistry and Chemical Engineering, Qiqihar University, Qiqihar, 161006, China; 2 Changchun Center of Mass Spectrometry & Chemical Biology Laboratory, Changchun Institute of Applied Chemistry, Chinese Academy of Sciences, Changchun, 130022, China; 3 Changchun University of Chinese Medicine, Changchun, 130117, China; University of Edinburgh, UNITED KINGDOM

## Abstract

This study attempted to clarify the material basis for the detoxification of *Rhizoma Zingiberis* (*RZ*) on aconitine, an analgesic drug, by quantitatively assessing the influence of *RZ* on the *in vitro* intestinal concentration of aconitine using an everted gut sac model and by qualitatively identifying the components in the *RZ* extract. To quantify aconitine in rat everted gut sacs, both an accurate processing method and a sensitive detection method were required. We developed a three-step sample processing method to protect the components from decomposition and applied ultra-performance liquid chromatography coupled with triple quadrupole mass spectrometry (UPLC/TQMS) to quantify aconitine, glucose and digoxin. In addition, ultra-performance liquid chromatography coupled with linear ion trap mass spectrometry (UPLC/ITMS) was applied to detect the potential antidotal components in the *RZ* extract. Finally, the *RZ* extract reduced the level of aconitine in everted gut sacs, and eleven gingerols were successfully identified, which could be considered potential antidotal components for aconitine. This study demonstrated the application of two UPLC/MS methods for analyzing the material basis for the reciprocity between Chinese medicine components in everted gut sacs.

## Introduction

Aconitine primarily exists in aconite tubers. These plants and their herbal formulations have been widely used in clinical applications, which include anti-inflammatory and analgesic functions [1–3]. However, the toxicity resulting from the main alkaloids in these plants, such as aconitine, cannot be neglected [4–7]. Processing and compatibility are the two main methods that can reduce the toxicity of aconite tubers through decreasing the content of toxic alkaloids [8, 9]. With respect to compatibility, the *in vivo* interaction of medicines must be considered. The intestinal tract is the main absorptive site for orally administered medicines, and interactions between medicines related to P-glycoproteins might affect their plasma concentration. Although previous studies have demonstrated that *Rhizoma Zingiberis* is a traditional Chinese medicine that can decrease the toxicity of aconitine [10], the material basis for the detoxification of *Rhizoma Zingiberis* has not yet been clarified. Furthermore, studies on the mechanism of aconitine toxicity [11–13] are currently more common than those on the detoxification mechanism. Therefore, this study attempted to elucidate the reason and the material basis for the detoxification of *Rhizoma Zingiberis* on aconitine using an everted gut sac model.

In this study, validating the methodology involved a processing method, and a test method was necessary to obtain a credible conclusion. Therefore, we developed a three-step processing method that consisted of freeze-drying, dissolving and centrifuging to separate inorganic salts from the chemical components and to prevent their degradation in the everted gut sac samples. The test methods in the current study included quantifying aconitine and digoxin, semi-quantifying glucose and qualitatively determining the components in *Rhizoma Zingiberis*. For the semi-quantification of glucose, we developed a UPLC/MS method as a substitute for the UV method to validate the viability of the everted gut sacs. UPLC coupled with quadrupole mass spectrometry was used to quantify aconitine and digoxin with low limits of quantification (LLOQ) of 8 and 16 ppb, respectively. In contrast, UPLC coupled with linear ion trap mass spectrometry was used to identify the components in *Rhizoma Zingiberis*.

P-glycoprotein is an important membrane transporter pump that mediates drug efflux in the intestinal epithelium [14,15]. This glycoprotein can protect cells from deleterious exogenous compounds by increasing the efflux of them, and it is related to the plasma concentration of numerous compounds. Therefore, it is necessary to determine the relationship between P-glycoprotein and aconitine as well as *Rhizoma Zingiberis* to elucidate the detoxification mechanism of *Rhizoma Zingiberis* on aconitine.

Verapamil [16] is an example of a P-glycoprotein inhibitor that can increase *in vivo* drug concentration by inhibiting P-gp activity. Digoxin is a ubiquitous substrate of P-gp [17, 18] that is commonly used to screen inhibitors or revulsants of P-gp. Previous studies conducted using a Caco-2 cell culture model indicated that aconitine was both a substrate and an inhibitor of P-glycoprotein and that there was a strong correlation between the absorption of aconitine and the activity of P-gp [19]. The everted gut sac model has been the most widely used model for investigating the absorption of drugs *in vitro*, other than the Caco-2 cell culture model, in recent studies [20–22].

In this study, a very simple and rapid sample processing method and methods for detecting glycoside, aconitine, digoxin and gingerols by applying UPLC/MS were developed. The developed methods were used to study the material basis for the detoxification effect of *Rhizoma Zingiberis* on aconitine using an everted rat gut sac model for the first time. The results are expected to be important for future in-depth studies.

## Materials and Methods

### Chemicals

The reference standards for aconitine and 6-gingerol were purchased from the Institute for the Control of Pharmaceutical and Biological Products of China (Beijing, China). *Rhizoma Zingiberis* was purchased from the Jilin Pharmacy (Changchun, China). Verapamil and digoxin were purchased from the Sigma Chemical Co. (St. Louis, MO, USA). Krebs-Ringer’s (K-R) solution [23] was composed of 1.4 g of glutamine, 7.8 g of sodium chloride, 0.35 g of potassium chloride, 0.32 g of sodium dihydrogen phosphate and 1.37 g of sodium bicarbonate dissolved in 1000 mL of distilled water. Methanol and acetonitrile were of HPLC grade. Dimethylsulfoxide (DMSO) was of analytical grade. Distilled water was prepared using a Millipore water purification system. Aconitine solutions were prepared with a high concentration, an intermediate concentration and a low concentration of 0.3, 0.03 and 0.003 mg/mL, respectively. A digoxin solution was prepared with a concentration of 0.8 mg/mL. A verapamil solution was prepared with a concentration of 1.0 mg/mL. A 10 mL aqueous extract of 10 g *Rhizoma Zingiberis* was prepared using the decocting method.

### Animals

Forty-three male Wister rats (200–220 *g*) were purchased from the Changchun Institute of Biological Products (Changchun, China). All of the rats were acclimated in the animal house under the following conditions: a temperature of 25°C and a humidity of 50% for seven diurnal cycles. During the seven days, food and water were provided *ad libitum*. After seven days of acclimation, the forty-three rats were divided into nine groups: the control group (three rats for detecting glucose), a high-concentration aconitine group (five rats), an intermediate-concentration aconitine group (five rats), a low-concentration aconitine group (five rats), an aconitine with *Rhizoma Zingiberis* extract group (five rats), an aconitine with verapamil group (five rats), a digoxin with *Rhizoma Zingiberis* extract group (five rats), an aconitine with 6-gingerol group (five rats) and a digoxin with 6-gingerol group (five rats).

### Preparation of the everted rat gut sacs

All of the rats were starved for 24 h before the experiments. The rats were sacrificed by decapitation, and the duodenum, jejunum, ileum and colon were immediately removed and placed in a 37°C K-R solution that was oxygenated (O_2_/CO_2_, 95%:5%). After washing in order with K-R solution at room temperature, the four intestinal segments were everted on a glass rod (3 mm in diameter) with one end of each clamped before filling with fresh oxygenated K-R solution. The intestines were then sealed with braided sutures. Each sac was placed in a 10 mL test tube that contained 8 mL of oxygenated drug-containing K-R solution at 37°C. Then, 0.1 mL of serosal fluid was collected at the appropriate time points, and an equal volume of K-R solution was added to the serosal side. Finally, the solution in the sacs was collected to accurately calculate the volume, and each sac was accurately weighed before and after being cut open. The area of each sac was measured. All of the samples were analyzed using the UPLC-MS method. After analysis, the amounts (ng/mm^2^) of absorbed aconitine and digoxin were calculated. Each experiment was repeated five times, and the presented data represent the means ± S.D. This study was conducted in accordance with the internationally accepted principles for laboratory animal use and care as found in the European Community guidelines.

### Validation of the viability of the everted rat gut sacs

**Fig 1:**
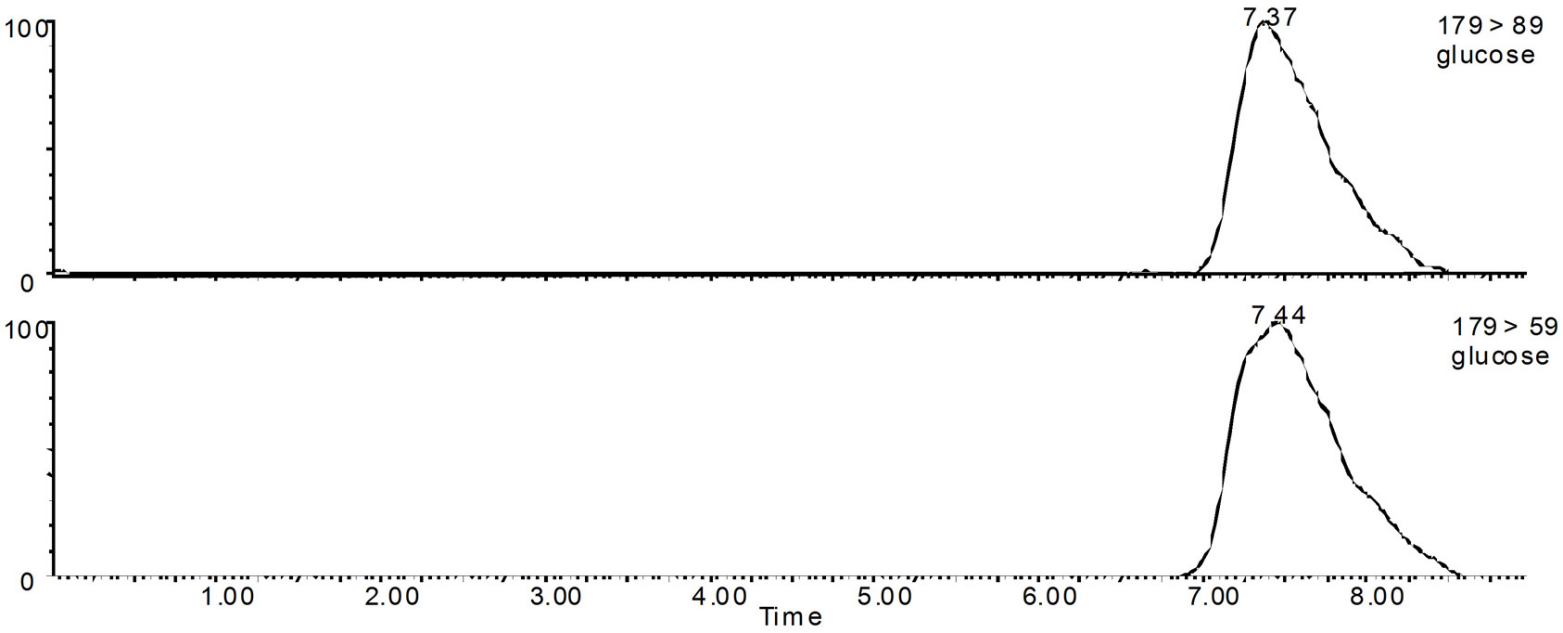
Multiple reaction monitoring (MRM) ion chromatogram of glucose.

To verify the integrity and viability of the gut sacs, glucose was collected from both the serosal side and the mucosal side of the rat gut sacs at a fixed time, and glucose was detected using a Waters UPLC/MS (Waters Co., USA), which is more accurate and sensitive than a UV-Vis detector. Separation was performed on a Hypersil NH column (4.6*150 mm, 25 μm, Elite Co., China). Although only glucose was available for detection in this part, the column separation was essential because large amounts of inorganic salts were present in the K-R nutrient solution. A mobile phase composed of 80% acetonitrile (A) and 20% distilled water (B) was used for isocratic elution. The retention time for glucose was 7.37 min. The chromatogram of glucose is shown in Fig 1. The peak area ratio of glucose in the serosal side to that in the mucosal side was used to validate the viability of the everted rat gut sacs. Because glucose was actively transported in the intestine, intact and active sacs concentrated glucose in the serosal side, and ratio of the glucose concentration in the serosal side to that in the mucosal side exhibited an increasing trend throughout the entire experiment.

### UPLC-MS detection of aconitine

**Fig 2:**
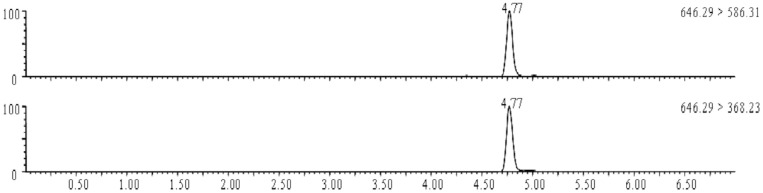
MRM ion chromatogram of aconitine.

The analytical UPLC-MS system consisted of an Acquity Ultra Performance LC with a Xevo TQ mass spectrometer as the detector, and it was equipped with an electrospray source in the positive ion mode (Waters Co., USA). The mass spectrometry conditions used to measure aconitine are shown in Table 1. Separation was performed on a BEH Extend-C18 column (2.1*50 mm, 1.7 μm, Agilent Co., USA). The mobile phase was composed of methanol mixed with acetonitrile in equal volumes (A) and an aqueous solution of ammonium acetate at pH 10.5 (B). The gradient elution conditions were as follows: 0–1 min 10–43% A, 2–4.5 min 55–100% A, and 5–7 min 10% A. The flow rate was 0.3 mL/min. The column temperature was 35°C throughout the entire detection process. All of the samples were freeze-dried after being collected from the serosal side, and the residue was dissolved with 0.1 mL of the mobile phase (A) prior to injecting 5 μL of each sample into the UPLC system. The UPLC retention time of aconitine was 4.77 min. The MRM ion chromatogram of aconitine is shown in Fig 2.

**Table 1:** Mass spectrometry conditions of detected compounds in this paper.

Detected compounds	Ion mode	scan mode	cone voltage(V)	collision energy(V)	Diagnosed ions(m/z)	capillary voltage(kV)	desolvation temp(°C)	Desolvation gas(L/h)	collision gas (mL/min)
aconitine	positive	MRM	54	34, 42	586.31,368.23	3.00	350	700	0.15
digoxin	negtive	MRM	50	35, 45	649.64, 475.35	3.00	350	700	0.15
glucose	negtive	MRM	10	15, 15	59, 89	2.5	350	700	0.15
**Detected compounds**	**Ion mode**	**scan mode**	**capillary voltage(V)**	**tube lens voltage(V)**	**source voltage(kV)**	**capillary temp(°C)**	**sheath gas (L/min)**	**auxiliary gas(L/min)**	
gingerols	positive	TIC	3	65	4	250	9.9	1.65	

### UPLC-MS detection of digoxin

**Fig 3:**
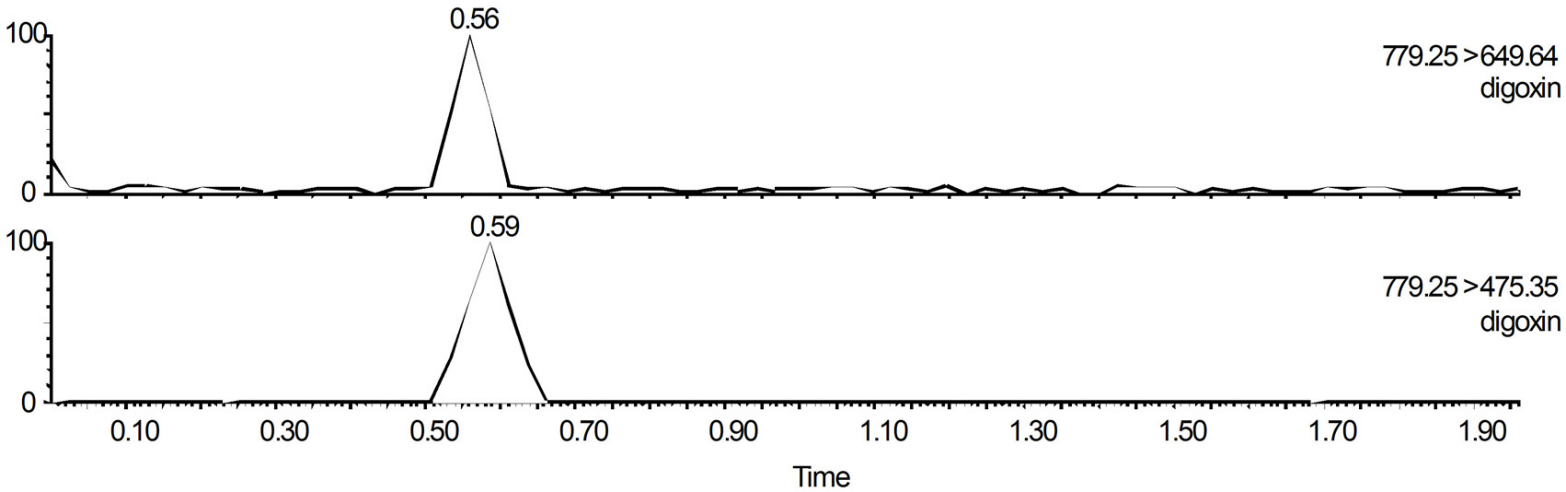
MRM ion chromatogram of digoxin.

Digoxin was detected using the same UPLC-MS system and chromatographic column used for detecting aconitine. However, the negative ion mode was applied to acquire the best signal for digoxin. The mass spectrometry conditions used to measure digoxin are shown in Table 1. Mobile phases composed of methanol mixed with acetonitrile in equal volumes (A) and water (B) were used for isocratic elution at the volume ratio of 70% (A) to 30% (A). The flow rate was 0.3 mL/min. The column temperature was 30°C throughout the entire detection process. All of the samples were freeze-dried after being collected from the serosal side, and the residue was dissolved with 50 μL of dimethyl sulfoxide and 50 μL of mobile solvent prior to injecting 5 μl of each sample into the UPLC system. The UPLC retention time of digoxin was 0.56 min. The chromatogram of digoxin is shown in Fig 3.

### UPLC-MS detection of gingerols

The gingerols in the *Rhizoma Zingiberis* extract were detected using the UPLC/ESI-MS^n^ system. This system consists of an ACCELA 1250 Pump with an LTQ ion trap mass spectrometer as the detector, and it is equipped with an electrospray source in the positive ion mode, which is capable of analyzing ions up to *m/z* 2000 (Thermo Co. USA). The *m/z* scan range was set to 100–800. The mass spectrometry conditions used are shown in Table 1. The separation was performed on a BEH Extend-C18 column (3×50 mm, 1.7 μm, Agilent Co., USA). The mobile phase was composed of methanol (A) and water (B). The gradient elution conditions were 0–12 min 40–100% A, 12–15 min 100% A. The flow rate was 0.3 mL/min. The column temperature was 30°C throughout the entire detection process.

### Assay validation for aconitine and digoxin

The UPLC/MS method for the detection of aconitine and digoxin was validated in terms of selectivity, linearity, accuracy, limit of detection (LOD), low limit of quantification (LLOQ), matrix effect, extraction recovery, and stability. A calibration curve was constructed by correlating the peak area with the concentration of aconitine or digoxin spiked in K-R nutrient solution. No weighting was applied.

The selectivity was examined by detecting blank K-R nutrient solutions, which were useful only if no peaks appeared at the retention times of aconitine and digoxin. The inter-day and intra-day accuracies were evaluated by calculating the percent relative standard deviation (RSD %) of the concentration of the quality control (QC) samples on the same day or over three days. The matrix effect was determined by comparing the concentrations of samples with standard solution added into a blank K-R solution after extraction with QC samples. The extraction recovery was calculated by comparing the concentration of normally extracted samples with that of the QC samples. The stability was evaluated by calculating the RSD % of the concentration of the quality control (QC) samples at 0 h, 24 h and 48 h. The accuracy, matrix effect, extraction recovery and stability were all replicated by five QC samples at three levels. The LOD and LLOQ were the concentrations of QC samples with peak signal-to-noise ratios (S/N) of at least 3 and 10, respectively.

### Ethics statement

This study was approved by the Ethics Committee of the Affiliated Hospital to Changchun University of Chinese Medicine and complied with all national and international guidelines on research involving animals. All of the rats were sacrificed by decapitation before the intestinal sacs were removed.

## Results

### Evaluation of methodology

Because there were numerous inorganic salts that were harmful to the mass spectrometer in the everted sac samples, dechloridation was the first consideration when we developed the processing method. We applied lyophilization as the first step for prolonging the resting period of the samples to avoid the decomposition of unstable components. The inorganic salts could not be dissolved in organic solvents such as methanol, whereas aconitine was able to be dissolved in methanol. Therefore, the second step consisted of dissolving aconitine in methanol. For digoxin, because it only could be dissolved in dimethyl sulfoxide (DMSO) among the tested solvents, which included methanol, ethanol, acetonitrile, and distilled water, the residue of the freeze-dried samples was dissolved with the same volumes of DMSO and methanol. The third step consisted of centrifuging, and 5 μL of the supernatant liquid was injected into the UPLC/MS.

**Fig 4:**
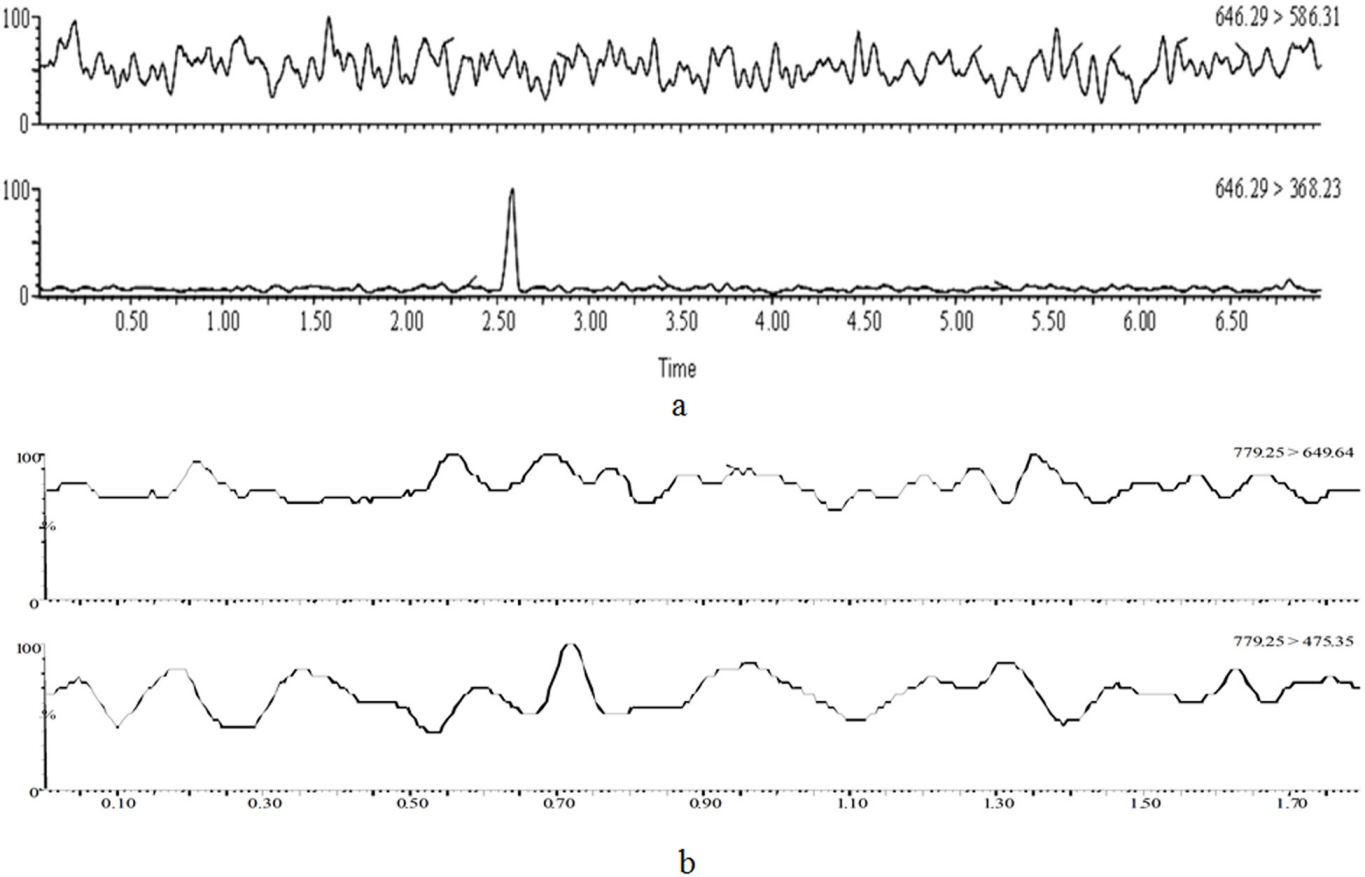
MRM ion chromatogram of aconitine(a) and digoxin(b) in blank intestinal nutritious solution.

The multiple reaction monitoring (MRM) ion chromatogram of the blank K-R nutrient solution is shown in Fig 4. We confirmed that the blank K-R nutrient solution did not interfere with the quantification of aconitine and digoxin.

The calibration curves for aconitine and digoxin were y = 164.7x–26455 and y = 0.1856x+17.12 at the linearity ranges of 1.813 to 18130 ng/mL and 2.048 to 32000 ng/mL, respectively.

The accuracy, matrix effect and extraction recovery, and stability are shown in Tables 2, 3 and 4. The RSD % of both the accuracy and stability were less than 15%, which was in the acceptable range and indicated that the repeatability of processing and the analytical method were adequate, and both aconitine and digoxin were stable for 48 h. The matrix effect and extraction recovery were between 85% and 105%. The LOD and LLOQ of aconitine were 0.03626 and 8 ppb, respectively, and these data for digoxin were 0.09065 and 16 ppb, respectively.

**Table 2:** Inter-day and intra-day precisions of aconitine and digoxin.

components	Added (ng/mL)	Found (ng/mL)	Inter-day RSD%	Intra-day RSD%	RE%
	14504	13018	3.450	1.405	10.24
aconitine	1133	1171	0.2911	10,62	3.375
	226.6	220.4	4.789	3.365	2.762
	16000	17231	7.735	3.995	7.694
digoxin	3200	3292	9.853	8.178	2.882
	128	125.8	14.17	7.657	1.709

**Table 3:** Extracted recovery and matrix effects of aconitine and digoxin.

components	Added (ng/mL)	Matrix effect (%)	Extracted recovery (%)
aconitine	14504	99.36±8.421	100.7±9.285
	1133	88.69±4.950	98.13±1.949
	226.6	101.7±3.763	102.7±1.004
digoxin	16000	90.77±3.157	89.38±4.530
	3200	101.2±3.921	97.56±9.564
	128	93.56±3.386	86.97±4.436

**Table 4:** Stabilities of aconitine and digoxin.

		RSD%	
component	High concentration	Middle concentration	Low concentration
aconitine	10.04	11.72	2.448
digoxin	1.336	4.913	2.036

### Viability of the gut sacs

**Fig 5:**
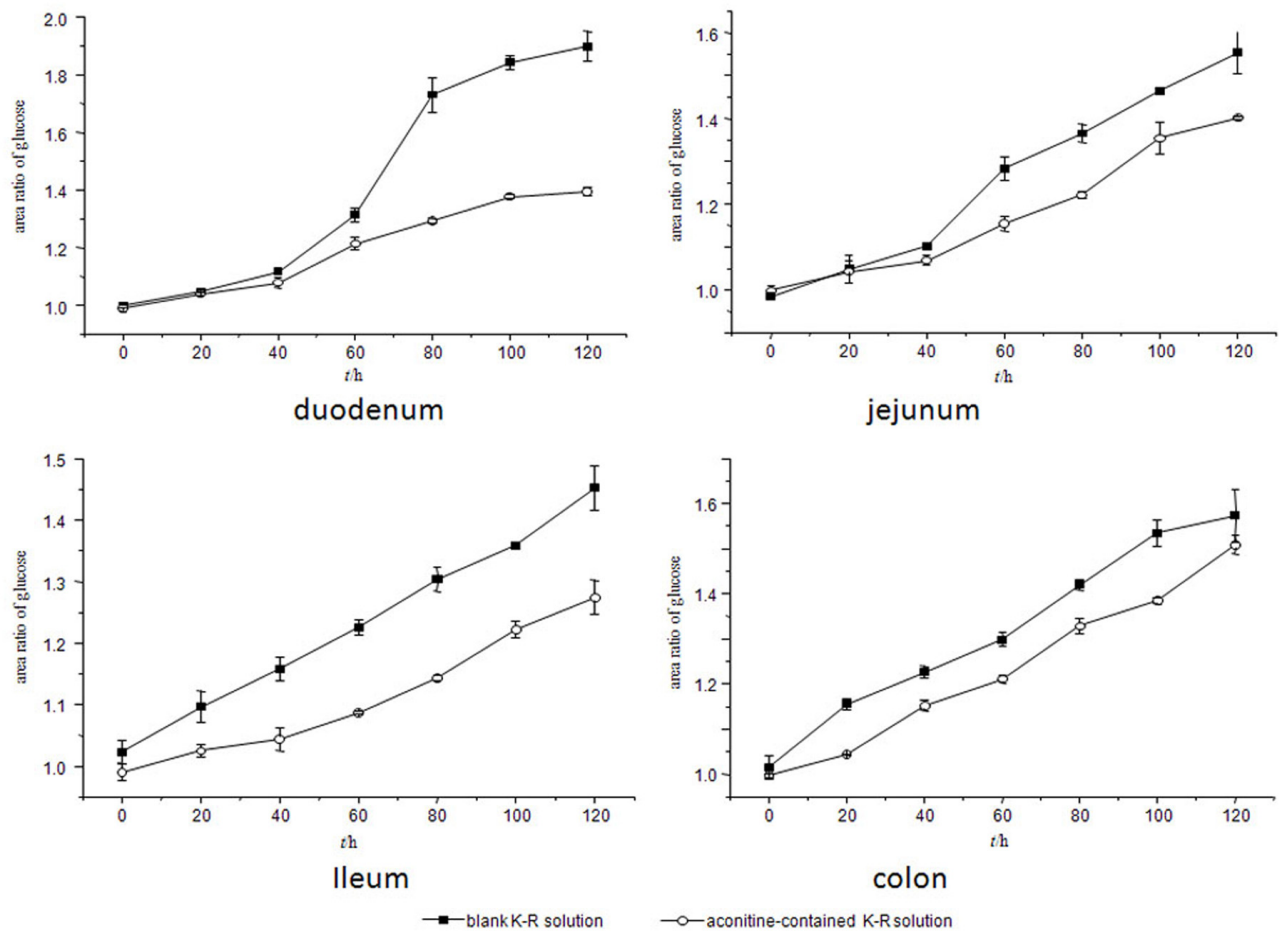
Ratios of glucose content in the serosal side to that in the mucosal side in different sacs.

Fig 5 (data for this figure are from parallel three rats samples) shows that both in the absence and presence of aconitine, the ratio of the glucose content in the serosal side to that in the mucosal side increased as the incubation time increased to 120 min, which indicated that the tissues of the gut sacs were viable and that aconitine did not induce toxicity in the gut sac tissue.

### Absorptive behavior of aconitine in the rat gut sacs

The intestinal kinetic absorptive behavior of aconitine was investigated in the gut sac model cultured in K-R nutrient solution for 120 min. The accumulative absorbed dose (Q) of aconitine was calculated for use in zero-level, primary and Higuchi equation fitting. Then, the determination coefficient (R^2^) of the equation was set as the evaluation criterion for the quality of the fit. The results indicated that the zero-level fitting equation was better than the other two equations, with R^2^>0.9. The zero-level fitting equation for different concentrations of aconitine in different rat gut sacs is shown in Table 5. The slope of the fitting equation represents the absorption rate constant (ka). Table 5 shows that ka increased as the concentration of aconitine in each gut sac increased, which indicated that the absorption of aconitine in the gut sac depended on the initial concentration of aconitine.

**Table 5:** Zero-level fitting equations for different concentrations of aconitine in different rat gut sacs.

Rat gut sacs	Concentratio(/ng·mL^-1)^	Fitting equation	Determination coefficient (R^2^)
	7500	Q = 3.528t	0.9338
duodenum	750	Q = 0.05406t	0.9834
	75	Q = 0.02546t	0.9715
	7500	Q = 3.2531t	0.9462
jejunum	750	Q = 0.2028t	0.9345
	75	Q = 0.02977t	0.9498
	7500	Q = 2.529t	0.9455
ileum	750	Q = 0.2906t	0.9688
	75	Q = 0.01294t	0.9188
	7500	Q = 2.000t	0.9695
colon	750	Q = 0.1302t	0.9548
	75	Q = 0.01937t	0.9521

### Effect of the *Rhizoma Zingiberis* extract on the level of aconitine in the rat gut sacs

To study the effect of the *Rhizoma Zingiberis* extract on the concentration of aconitine in the rat gut sacs, the apparent osmotic coefficient (Papp) was calculated using the equation Papp = [V/(A·C_0_)]·dC/dt, where V represents the fluid volume in the serosal side, A represents the superficial area of the gut sac, and C_0_ represents the initial concentration of aconitine outside the gut sac. The calculated Papp value of aconitine is shown in Table 6. The results of a variance analysis for P_0_ indicated that different gut sacs influenced the level of aconitine, that the P_0_ value of aconitine for the jejunum sac was greater than in the other three sacs, and that the value for the colon sac was smallest among the four sacs. In addition, the P_1_ value of aconitine for each gut sac decreased after the *Rhizoma Zingiberis* extract was added, and the decrease in the duodenum was most significant, which can be observed from its lowest ER value. These results indicate that the *Rhizoma Zingiberis* extract reduced the level of aconitine in the rat gut sacs, and this reduction was greatest in the duodenum; however, the mechanism could not be elucidated from the above results. Therefore, further experiments were performed to elucidate the possible reason for the reduction in the level of aconitine by the *Rhizoma Zingiberis* extract.

**Table 6:** Effect of the *Rhizoma Zingiberis* extract on the Papp value of aconitine.

groups	duodenum	jejunum	ileum	colon
high aconitine concentration (P_0_)	(4.111±0.3898)E-05	(4.751±0.4762)E-05	(4.386±0.1086)E-05	(3.506±0.03262)E-05
high aconitine concentration with the extract of *Rhizoma Zingiberis* added (P_1_)	(1.111±0.1150)E-05	(1.764±0.1251)E-05	(1.585±0.06571)E-05	(2.347±0.1634)E-05
high aconitine concentration with 6-gingerol added (P_2_)	(1.481±0.1242)E-05	(1.781±0.1923)E-05	(1.519±0.09117)E-05	(1.530±0.1401)E-05
ER(P_1_/P_0_)	0.2703	0.3942	0.3615	0.6695
ER(P_2_/P_0_)	0.3602	0.3979	0.3464	0.4363

### Effect of a P-glycoprotein inhibitor on the level of aconitine in the rat gut sacs

**Fig 6:**
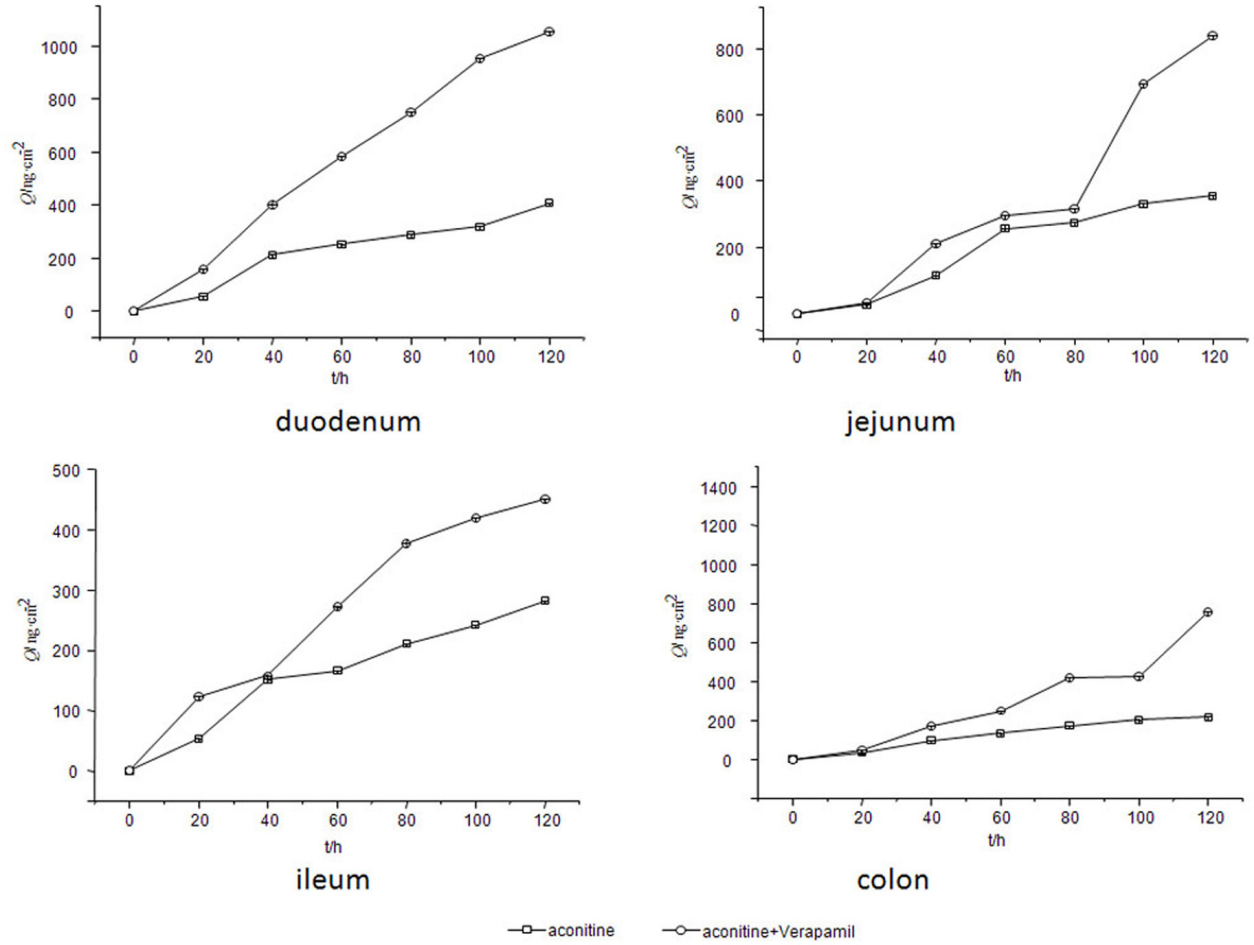
Effect of verapamil on the absorptive profile of aconitine.

P-gp is an important glycoprotein in the intestinal mucous membrane epithelia. Because it can help pump medicine from the intestinal serosal side to the mucosal side, P-gp has a negative effect on the level of a majority of medicines. However, for toxic substances, P-glycoprotein might play a positive role. To determine the potential role of intestinal P-gp in the dynamic level of aconitine, verapamil (a known P-gp inhibitor), was added to a K-R solution that contained a high concentration of aconitine. As shown in Fig 6 (data for this figure are from parallel five rats samples), the content of aconitine increased when verapamil was added into every gut sac; in other words, the inhibition of intestinal P-gp by verapamil significantly increased the aconitine content in the rat intestine. Therefore, it can be deduced that aconitine is a potential substrate of P-glycoprotein.

### Effect of the *Rhizoma Zingiberis* extract on the level of P-gp substrates

**Fig 7:**
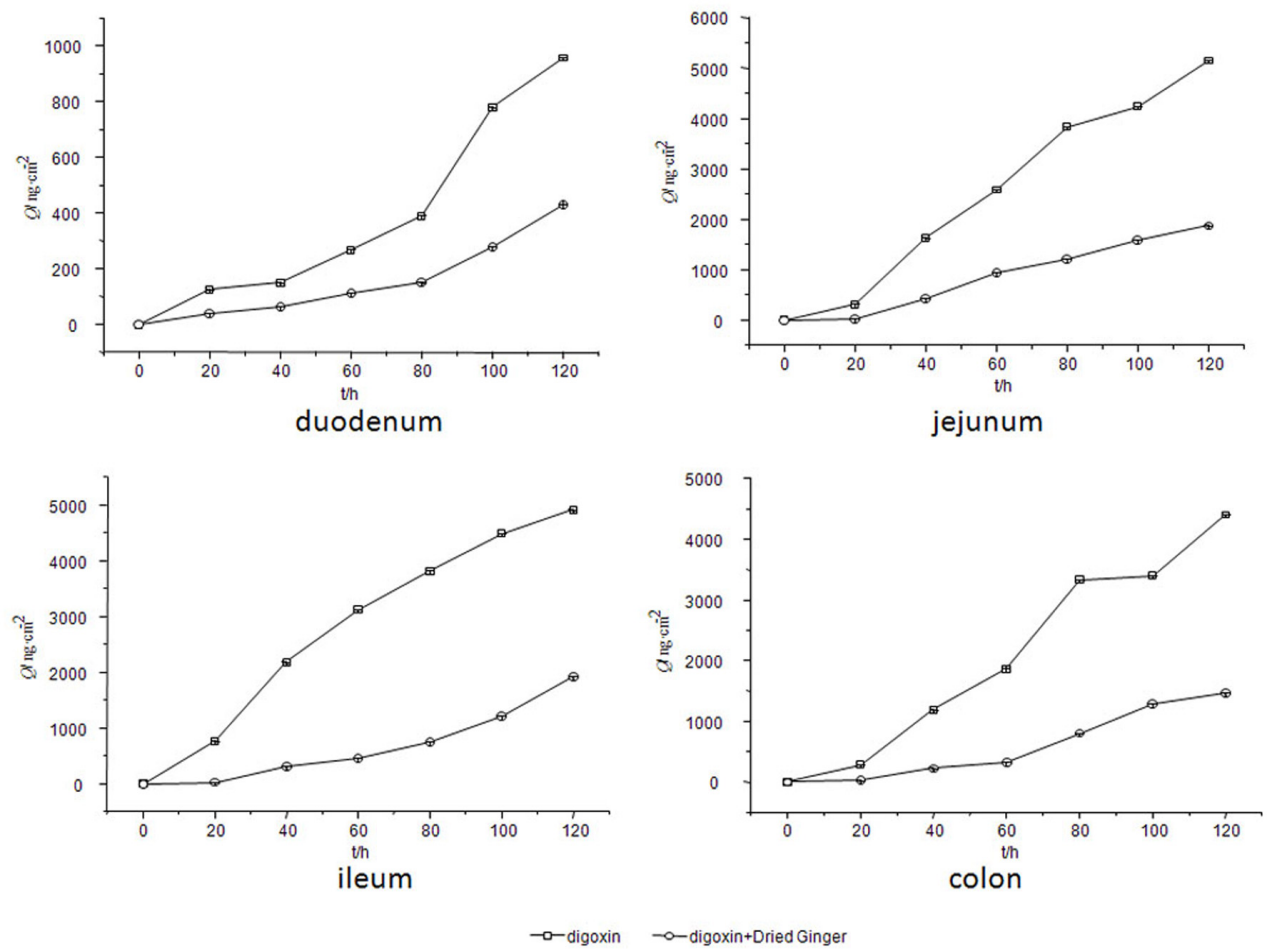
Effect of the *Rhizoma Zingiberis* extract on the absorptive profile of digoxin.

Because digoxin is a well-known substrate of P-glycoprotein, it was selected to be incubated with the *Rhizoma Zingiberis* extract to determine whether this extract could alter the level of digoxin. As shown in Fig 7 (data for this figure are from parallel five rats samples), the digoxin content decreased in every gut sac when the *Rhizoma Zingiberis* extract was added; in other words, the *Rhizoma Zingiberis* extract decreased digoxin level in the rat intestine. Therefore, it can be deduced that the *Rhizoma Zingiberis* extract might be an inducer of P-glycoprotein. Furthermore, it can be deduced that the *Rhizoma Zingiberis* extract might decrease the conitine content by activating P-glycoprotein.

### Detection of gingerols in the Rhizoma Zingiberis extract

The above results demonstrated that the *Rhizoma Zingiberis* extract reduced the level of aconitine in the rat gut sacs by inducing intestinal P-gp; however, the material basis was not determined. Therefore, the following experiments were conducted to identify the main components of *Rhizoma Zingiberis* that affect aconitine levels in the rat gut sacs.

**Fig 8:**
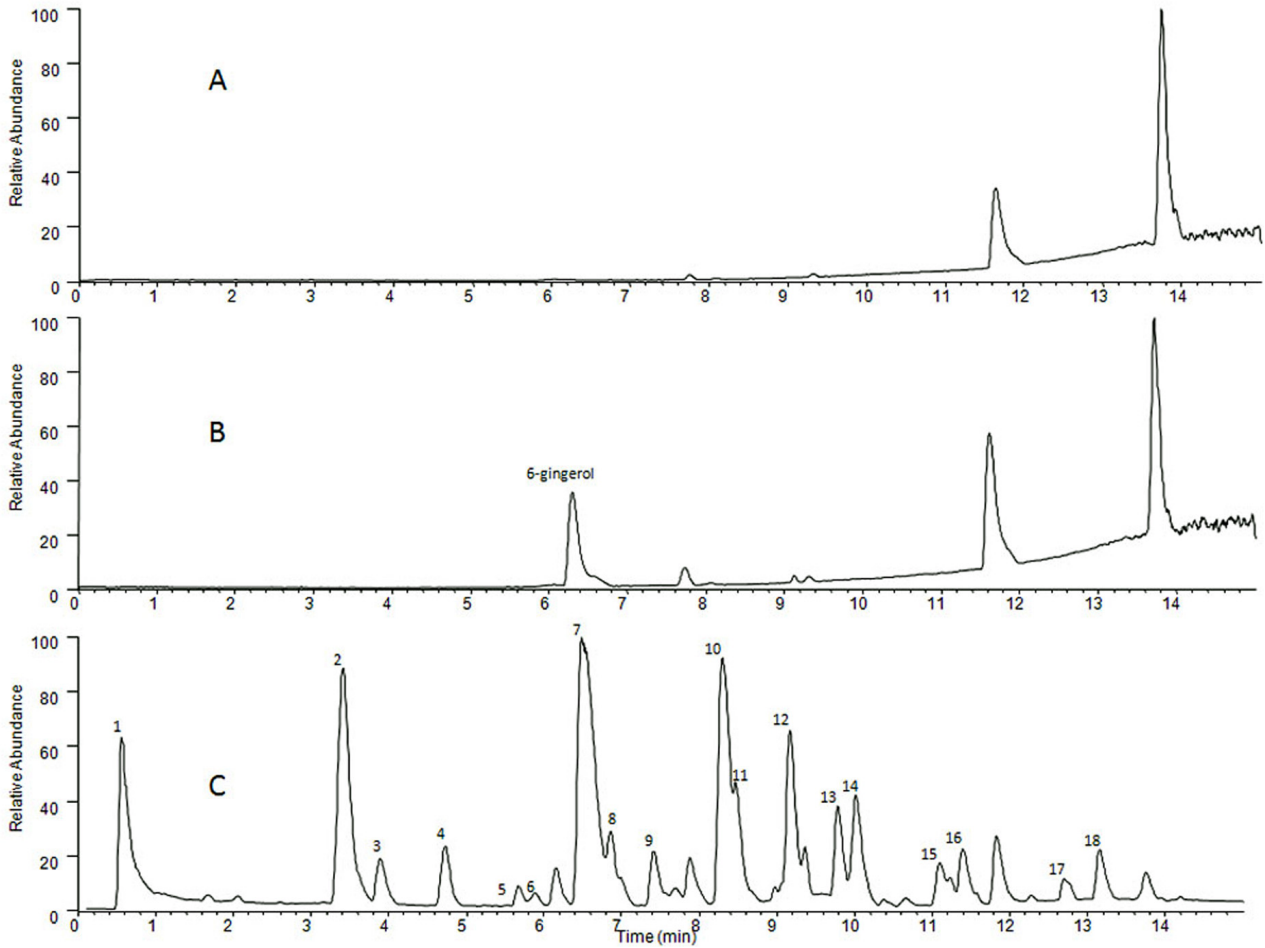
Base peak ion chromatogram of the extract of *Rhizoma Zingiberis*: (A) blank solvent, (B) 6-gingerol standard, and (C) the *Rhizoma Zingiberis* extract.

Previous reports have suggested that the major bioactive constituents in *Rhizoma Zingiberis* are gingerols [24–26]. In this section, the general components in the aqueous extract of *Rhizoma Zingiberis* were detected using UPLC/MS^n^, and the base peak chromatogram was shown in Fig 8. In Fig 8, eleven peaks were identified tentatively on the basis of their diagnostic ions and of a previous study [27], as shown in Table 7. 6-Gingerol, which was eluted at 6.50 min, presented the highest peak; thus, the effect of 6-gingerol on the intestinal level of aconitine was investigated.

**Table 7:** Preliminarily determined phenols in the aqueous extract of *Rhizoma Zingiberis*.

Peak No.	*t*_R_ (min)	[M+H]^+^ (*m/z*)	[M+H_2_O]^+^ (*m/z*)	[M+Na]^+^ (*m/z*)	[2M+Na]^+^ (*m/z*)	Identified phenols
1	0.57	267	—	289	—	4-gingerol
3	3.89	305	—	—	—	8-shogaol
5	5.66	395	—	—	—	Me-12-gingediol’isomer
7	6.50	295	312	317	611	6-gingerol
9	7.41	325	—	347	—	8-gingediol
10	8.30	277	294	299	575	6-shogaol
11	8.46	323	340	345	667	8-gingerol
12	9.15	—	398	403	—	12-gingediol
13	9.77	—	412	417	—	Me-12-gingediol
14	10.01	351	368	373	723	10-gingerol
16	11.38	333	350	355	687	10-shogaol

### Effect of 6-gingerol on the level of aconitine in the rat gut sacs

The experiment described in this section was conducted under the same conditions as decribed in the “Effect of the *Rhizoma Zingiberis* extract on the level of aconitine in the rat gut sacs” section. The calculated Papp value of aconitine is shown in Table 6. As shown in Table 6, the P_2_ value is smaller than the P_0_ value in each gut sac, and the P_2_ value for the ileum was the smallest among the four gut sacs. From the above results, it could be concluded that 6-gingerol, as well as other gingerols in the *Rhizoma Zingiberis* extract, contributed to the decreased effect of the extract on the aconitine level.

### Effect of 6-gingerol on the level of digoxin in the rat gut sacs

**Fig 9:**
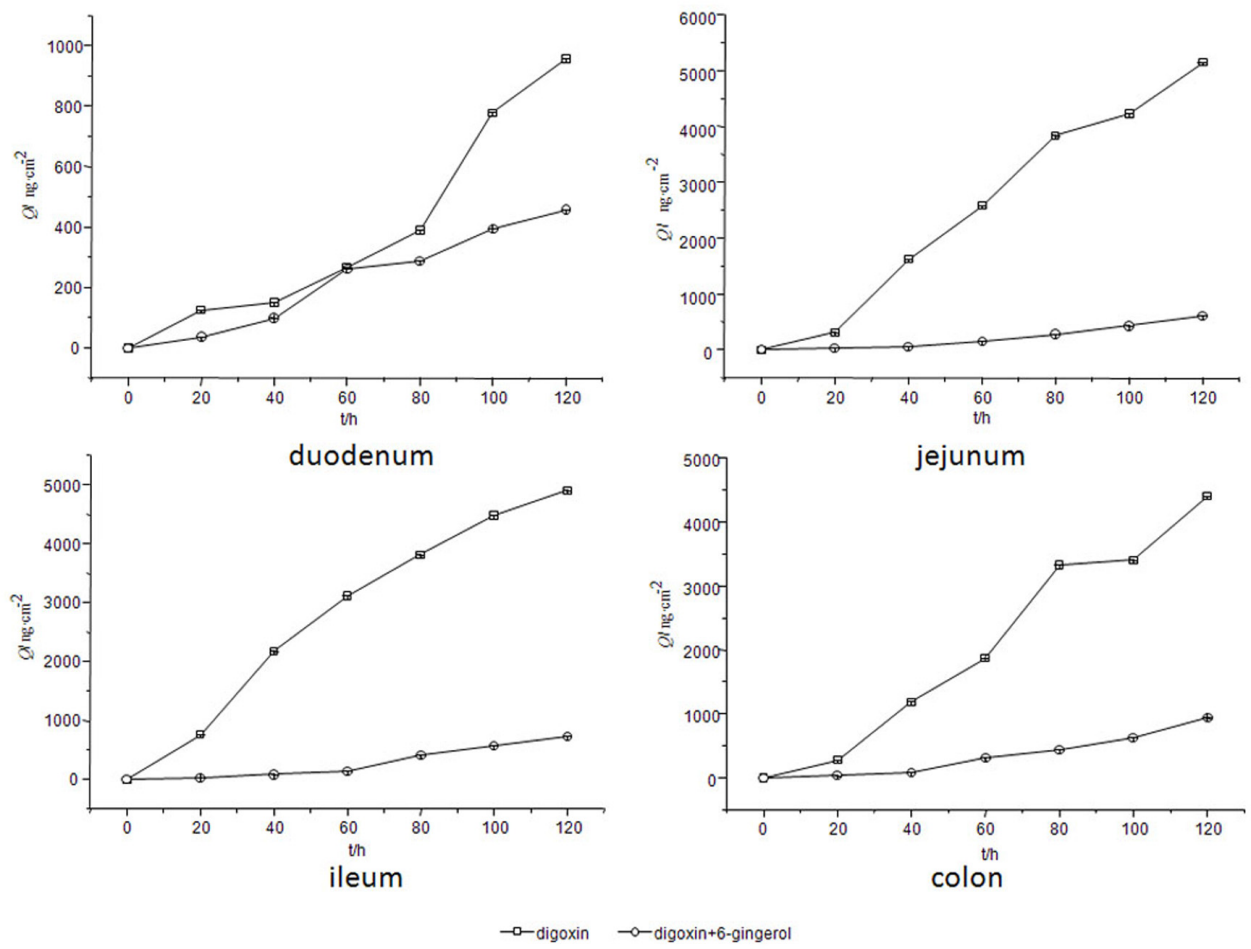
Effect of 6-gingerol on the absorptive profile of digoxin.

To study the mechanism by which the effect of 6-gingerol on the aconitine level was reduced, 6-gingerol and digoxin were co-incubated. The effect of 6-gingerol on the digoxin concent in various rat gut sacs is clearly visible in Fig 9 (data for this figure are from parallel five rats samples). The obtained results show that the digoxin content in the rat gut sacs was decreased after adding 6-gingerol. Therefore, it can be concluded that 6-gingerol is a potential revulsant of P-gp; together with the previous conclusion, this finding indicates that 6-gingerol reduced the level of aconitine by inducing P-gp.

## Discussion

Aconitine is a well-known toxic compound that has been the focus of many previous studies. Previous studies have shown that various traditional Chinese medicines, such as *Radix Glycyrrhizae* and *Rhizoma Zingiberis* can decrease the toxicity of aconitine. The content of aconitine in the co-decoction of *Rhizoma Zingiberis* with *Radix Aconiti Lateralis Preparata* was not less than that in the single decoction of *Radix Aconiti Lateralis Preparata*, whereas the LD_50_ value of the co-decoction was larger than that of the single *Radix Aconiti Lateralis* decoction [28]. Therefore, it was hypothesized that *Rhizoma Zingiberis* might reduce the plasma concentration of aconitine. Based on the above proposal, this study was conducted.

Drugs are absorbed into the blood stream mainly in the intestine; therefore, everted rat gut sacs were studied to determine the methanism by which *Rhizoma Zingiberis* affects aconitine transport and to determine the main extract components responsible. The findings demonstrate that aconitine is a potential substrate of intestinal P-gp and that the *Rhizoma Zingiberis* extract is a potential revulsant of P-glycoprotin; in addition, the *Rhizoma Zingiberis* extract reduced the permeation of aconitine by inducing P-glycoprotein, and gingerols were the main *Rhizoma Zingiberis* components responsible for the effect of *Rhizoma Zingiberis* on aconitine transport in the intestine. Early studies showed that the substrate, which comprised at least one basic nitrogen atom and an aromatic ring containing benzoyl groups, could be combined with P-glycoprotein [29]. Therefore, the structure of aconitine indicates that this compound is a substrate for P-glycoprotein, which is consistent with the results obtained in this study. Previous studies showed that the rate of transport of the P-glycoprotein substrate is related to the nature of the hydrogen bonds formed between the substrate and P-glycoprotein (stronger hydrogen bonds result in slower transport rates) [30]. Large numbers of phenols that contain hydroxy groups are present in the molecular structures of *Rhizoma Zingiberis* extract components; these hydroxy groups competed with aconitine to form hydrogen bonds with P-gp. Thus, the induction of P-gp by gingerols present in *Rhizoma Zingiberis* might be related to a weakening of the hydrogen bonds that are formed between aconitine and P-glycoprotein.

## Conclusion

This paper demonstrated an effective reduction in the toxicity of aconitine, namely, the *Rhizoma Zingiberis* extract, reduced the intestinal aconitine level by inducing intestinal P-gp, which will be verified by our further studies on acute toxicity test. This conclusion is consistent with previous studies using the Caco-2 cell culture model. Moreover, the material basis for the detoxification of *Rhizoma Zingiberis* on aconitine might be all of the gingerols involved in this study. This study provided a strategy for identifying new P-gp inducers and activators to search for new antidotes to be used in intoxication caused by P-gp sbustrates.
